# Advances in the application of novel smart hydrogels for periodontal tissue regeneration

**DOI:** 10.3389/fbioe.2026.1820467

**Published:** 2026-06-03

**Authors:** Xue Wang, Xin Mu, Jiqing Zhao, MingZhen Tan, Yujie Han, Hualong Gao, Nan Zhang, Kun Liu

**Affiliations:** 1 School of Stomatology, Shandong Second Medical University, Weifang, Shandong, China; 2 Shandong Provincial Key Medical and Health Laboratory of Stem Cell and Regenerative Medicine Translation, Liaocheng, Shandong, China; 3 Stem Cell and Regenerative Medicine Laboratory, Liaocheng People’s Hospital, Liaocheng, Shandong, China; 4 School of Stomatology, Shandong First Medical University, Jinan, Shandong, China; 5 Department of Stomatology, Liaocheng People’s Hospital, Liaocheng, Shandong, China

**Keywords:** hydrogels, matrix metalloproteinases (MMP), metal-organic frameworks (MOFs), periodontal regeneration, periodontitis, reactive oxygen species (ROS)

## Abstract

Periodontal disease is a persistent inflammatory condition caused by pathogenic microorganisms, primarily characterized by the progressive destruction of the tooth-supporting structures (namely, the periodontal ligament, cementum, and alveolar bone) as well as the associated gingival tissues. Hydrogel matrices, distinguished by their superior cytocompatibility profiles, are increasingly recognized as viable therapeutic scaffolds for orchestrating periodontal tissue repair and functional restoration. This review outlines the fundamental requirements for hydrogel-based periodontal regeneration and highlights recent advances in smart, stimuli-responsive hydrogels responsive to endogenous stimuli (reactive oxygen species (ROS), matrix metalloproteinases (MMPs)) and exogenous stimuli (temperature, light, electric fields, pH). We describe innovative fabrication strategies, underlying responsive mechanisms, and associated molecular signaling pathways. Furthermore, we critically review probiotic-hydrogel composites that modulate oral microbiota homeostasis to construct immune microenvironments favorable for bone regeneration, as well as metal-organic framework (MOF)-based hydrogels achieving multifunctional therapeutic effects including antimicrobial, antioxidant, and osteogenic activities through sustained metal ion release. This review presents diverse design strategies for developing novel intelligent functional materials and establishes a theoretical foundation for precision therapeutic approaches against periodontitis.

## 1 Introduction

**FIGURE 1:**
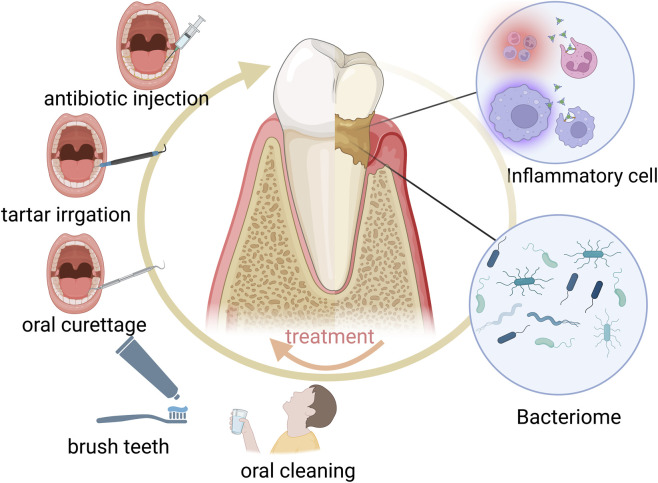
Conventional therapeutic strategies for periodontal disease: Combined antibiotic injection, dental calculus irrigation, and oral scaling and root planing to eliminate infectious foci, supplemented by daily oral hygiene and microbiota modulation, reducing inflammatory cell infiltration and optimizing the microenvironment to promote tissue regeneration. Created in https://BioRender.com.

Alveolar bone resorption resulting from periodontitis typically presents irregular morphologies. Conventional therapeutic modalities primarily encompass supragingival scaling and subgingival root planing. However, adjunctive periodontal surgery becomes necessary when increasing pocket depths accompany tissue destruction. Complete eradication of subgingival infectious sites and effective management of periodontal pockets represent critical steps in arresting disease progression. Nevertheless, achieving efficient and sustained delivery of therapeutic agents at lesion sites during such localized interventions remains a significant challenge (Santos et al., 2023; Wang and Gao, 2024) (Figure 1). Injectable hydrogels have emerged as promising research candidates in this field owing to their excellent biocompatibility and biodegradability. Their distinctive sol-gel transition characteristics enable *in situ* gelation following a single minimally invasive injection, thereby avoiding repeated invasive procedures while providing scaffolds for bone tissue regeneration (Alamir et al., 2023).

Hydrogels constitute three-dimensional hydrophilic polymer architectures stabilized through covalent or non-covalent crosslinking modalities (Hameed et al., 2024). These materials possess the capacity to imbibe and retain significant quantities of aqueous media without compromising their structural coherence. Their elevated water content provides desirable flexibility and excellent biocompatibility (Radulescu et al., 2022), while their osmotic pressure closely matches that of the extracellular matrix (ECM), thereby facilitating cell adhesion, proliferation, and nutrient exchange (Siddiqui et al., 2021). However, ideal hydrogels intended for periodontal therapy must satisfy strict multifunctional criteria including appropriate porosity; favorable mechanical properties; interconnected porous architectures to enable controlled drug release; *in vivo* biodegradability with non-toxic, non-inflammatory degradation byproducts; degradation kinetics matching drug release profiles to ensure sustained therapeutic efficacy; adequate tissue adhesiveness to prevent displacement; and integrated antimicrobial and anti-inflammatory functionalities to modulate the complex microenvironment characteristic of periodontitis (Sun et al., 2024). This review critically reviews the applications of hydrogels in periodontal tissue regeneration, describes critical design parameters for hydrogel development, summarizes advances in conventional hydrogel systems for periodontal tissue engineering, and integrates recent breakthroughs in next-generation smart hydrogels, thereby providing a comprehensive reference framework for periodontal regenerative strategies.

## 2 Design considerations for hydrogels in periodontal tissue regeneration

### 2.1 Appropriate porosity

The pore architecture of hydrogels constitutes a core engineering parameter determining the success or failure of periodontal tissue regeneration, with its influence extending throughout the entire process of mass transport, cellular behavior modulation, and mechanical support (Dos Santos et al., 2024). First, high porosity and interconnectivity are prerequisites for tissue ingrowth and mass transport: Koch et al. demonstrated that monocomponent P11-4/8 self-assembling peptide hydrogels, exhibiting a porosity of 46%, significantly enhanced cell adhesion and proliferation (Koch et al., 2018), while scaffolds prepared by Xu et al. with approximately 80% porosity provided a favorable microenvironment for stem cells and upregulated osteogenic gene expression (Xu et al., 2021). Second, surface and internal pores of hydrogel scaffolds exhibit distinct structural and functional specialization; leveraging this pore compartmentalization, researchers have engineered multifunctional scaffolds for periodontal regeneration, exemplified by Janus-structured hydrogels with a dense surface resisting bacterial colonization and a porous layer supporting osteogenesis (Huang et al., 2024), as well as bilayer hydrogel membranes developed by dos Santos et al., wherein a dense layer prevents soft tissue ingrowth and a honeycomb layer promotes bone regeneration (Dos Santos et al., 2024). Furthermore, pore architecture exerts a decisive influence on the diffusion of nutrients and oxygen, with porosity, pore size, and pore interconnectivity collectively governing internal mass transport efficiency. Tang et al. confirmed that macroporous structures enhance diffusion and promote vascularized bone regeneration (Tang et al., 2020). However, a complex trade-off relationship exists between porosity and mechanical properties: high porosity facilitates cell infiltration yet compromises mechanical strength. Ghasemzaie et al. systematically modulated hydrogel porosity using microbubble technology, revealing that porous PEGDA hydrogels exhibited significantly lower compressive moduli than dense controls, thereby elucidating a tunable trade-off between porosity and stiffness (Ghasemzaie et al., 2026). Nevertheless, this relationship can be optimized through nanomaterial incorporation. Hou et al. constructed porous cryogels with high modulus (>400 kPa) and macroporous architecture by regulating silk fibroin nanofiber concentration, achieving an optimized integration of mechanical strength and porosity, and validated their efficacy in vascularized bone repair in a rat femoral defect model (Hou et al., 2023). In summary, the precise balance among pore interconnectivity, hierarchical functionality, diffusion efficiency, and mechanical performance is critical to functional periodontal regeneration, and future efforts should systematically optimize pore architecture through advanced technologies such as 3D printing and freeze-casting to meet the complex demands of composite tissue regeneration.

### 2.2 Appropriate mechanical properties

To maintain the mechanical integrity of hydrogels during bone regeneration, mechanotransduction-the core biological process through which cells sense and respond to the mechanical microenvironment of the extracellular matrix, bridging mechanical signals at the solid-state physics level with the regulation of cellular behaviors at the molecular biology level-must be fully considered (Wu et al., 2024). Among numerous mechanical cues, the elastic modulus of the matrix represents the most characteristic key parameter; it is not merely a physical attribute, but rather a core regulatory factor that cells perceive and transduce into biochemical signals via the integrin-cytoskeleton pathway (Gan et al., 2023). Of particular importance, substantial evidence has demonstrated that matrices engineered with an elastic modulus matching that of native bone tissue actively direct the osteogenic lineage commitment of mesenchymal stem cells. Specifically, when hydrogel stiffness is matched to native bone tissue, mechanosensitive pathways such as YAP/TAZ are activated, thereby directionally inducing osteogenic differentiation (Moharrer, 2025). Accordingly, hydrogel mechanical properties can be optimized through tuning crosslinking density, incorporating nano-hydroxyapatite (HAP) or fibers, and constructing interpenetrating polymer networks (Wang Xiao et al., 2025). Studies have demonstrated that in NapAlen/HAP supramolecular systems, optimal HAP concentrations not only reinforce mechanical properties but also facilitate the formation of three-dimensional porous architectures, significantly promoting periodontal bone regeneration (Hu C. et al., 2023). Contemporary investigations have substantiated that nanocomposite hydrogels comprising 5% gelatin and 10% Laponite, when functionalized with small extracellular vesicles isolated from bone marrow-derived mesenchymal stromal cells (BMSC-sEVs), demonstrate prolonged release kinetics. These constructs markedly mitigate alveolar bone resorption, inflammatory cell infiltration, and collagen matrix degradation in rodent models, concurrently exhibiting favorable osteoconductivity and controllable degradation characteristics (Liu et al., 2021; Mohanto et al., 2023).

### 2.3 Appropriate degradation rate

The degradation rate of hydrogels represents a critical factor for periodontal tissue regeneration, requiring the maintenance of adequate mechanical support while regulating the dynamic homeostasis of the local microenvironment to guide controlled tissue regeneration, ultimately achieving synchronization between material degradation and *de novo* tissue formation (Wen et al., 2025). Insufficient degradation rates restrict the expansion space for regenerating bone tissue and delay the repair process (Gu et al., 2023); conversely, excessively rapid degradation leads to premature collapse of scaffold structures, compromising the stability of the regenerative microenvironment. However, the modulation of degradation rate is not the sole consideration-the biological effects of degradation byproducts also profoundly influence regenerative outcomes. Small molecular fragments or ionic species released during hydrogel degradation can directly or indirectly modulate macrophage polarization, osteoblast activity, and angiogenesis by altering local pH, osmotic pressure, and ionic concentration (Liu Y. et al., 2024); should acidic degradation products accumulate, they may elicit chronic inflammatory responses and compromise osseointegration (Gan et al., 2023; Rauner and Bozec, 2026). Parsaee et al. demonstrated that incorporating high concentrations of β-tricalcium phosphate (β-TCP) into chitosan/collagen composite scaffolds significantly reduces the disintegration rate, potentially due to enhanced electrostatic interactions between cationic species on the β-TCP surface and anionic groups within the matrix. Notably, Ca^2+^ and PO_4_^3-^ ions released during β-TCP degradation are inherently the core inorganic constituents of bone mineralization, capable of actively promoting osteogenic differentiation while retarding structural collapse, thereby exemplifying the synergistic design principle of “degradation kinetics-biological function of byproducts” (Parsaee et al., 2023). Therefore, the degradation kinetics of hydrogels must be precisely matched to the distinct stages of periodontal tissue regeneration.

### 2.4 Appropriate tissue adhesiveness, antimicrobial and anti-inflammatory properties

Hydrogels should also possess antibacterial, anti-inflammatory, and wet adhesion properties. Inspired by mussel adhesion mechanisms, researchers developed a tannic acid-silk fibroin-minocycline hydrochloride ternary system (TSM). This system can stably adhere in wet periodontal pockets for up to 28 days, achieving sustained drug release. It effectively inhibits periodontal pathogens, reduces inflammation and bone resorption, and shows good biocompatibility and clinical application potentia (Fan et al., 2025). Furthermore, a bifunctional antimicrobial architecture (designated CS-PA) was fabricated through the covalent conjugation of chitosan with antimicrobial peptide-functionalized polyethylene glycol. This matrix was subsequently impregnated with curcumin-encapsulated biodegradable nanospheres (CNP) to yield the CS-PA/CNP hybrid hydrogel. Localized delivery of this composite to the gingival crevice in a hypertensive periodontitis murine model demonstrated sustained immunosuppressive efficacy and marked amelioration of disease pathophysiology (Xu S. et al., 2023).

## 3 Conventional hydrogels for periodontal tissue regeneration

Because of their inherent three-dimensional network structures, excellent biocompatibility, and tunable physicochemical properties, conventional hydrogels were widely used early in periodontal tissue regeneration research to construct drug delivery systems, cell delivery platforms, and scaffolds or membranes, showing multiple functions in promoting tissue repair and regeneration.

### 3.1 Drug delivery systems

In local treatment of periodontitis, traditional drug delivery systems are affected by complex mechanical stress and continuous saliva flushing, making stable retention in periodontal pockets difficult. They are prone to displacement, detachment, or even accidental swallowing by patients; frequent administration also reduces patient comfort and compliance. In contrast, gel-based soft implant systems possess excellent plasticity, allowing minimally invasive filling of irregular periodontal cavities to achieve long-lasting drug retention and reduce pain and discomfort. These systems have become an important research focus for precision treatment of periodontitis (Li et al., 2023; Wang Y. et al., 2024).

Compared with conventional antibiotic therapy, hydrogels have emerged as ideal local antimicrobial carriers for periodontal applications owing to their controlled release, targeted delivery, and excellent biocompatibility (Mensah et al., 2023). Among these, the choice of drug release kinetics directly influences treatment outcomes: burst release rapidly controls acute infections but may compromise cell viability and induce bacterial resistance (Peng et al., 2024), whereas controlled release maintains sustained antibacterial activity at sub-inhibitory concentrations yet potentially delays infection control (Plastun et al., 2022; Wang X. et al., 2024). An optimal strategy therefore entails biphasic release-moderate early burst for infection containment followed by steady controlled release to promote regeneration (Ren et al., 2025; Yoon et al., 2025). Concurrently, the gradual release of degradation products facilitates the timely transition of macrophages from the M1 pro-inflammatory phenotype to the M2 reparative phenotype, whereas burst release may prolong the M1 state due to intense local stimulation, thereby compromising the repair outcome (Zhao et al., 2024). In addition, Zhang et al. developed an injectable alginate hydrogel that achieves slow drug release during degradation through co-crosslinking with the anti-inflammatory molecule spermidine. This system not only reduces inflammation but also inhibits osteoclast formation. Animal experiments show that this gel effectively promotes periodontal regeneration and collagen deposition. Notably, spermidine sustained-release circumvented polyamine cytotoxicity, corroborating the superiority of controlled release in balancing antimicrobial efficacy and cellular viability (Zhang et al., 2023). Such multifunctional drug delivery strategies provide important insights for periodontitis treatment and periodontal tissue regeneration; nevertheless, personalized optimization of release kinetics according to infection severity remains imperative to achieve an optimal equilibrium between cell preservation and pathogen eradication.

### 3.2 Platforms for cell and growth factor delivery

Stem cells constitute pivotal cellular substrates for periodontal regenerative strategies, attributable to their intrinsic capacity for self-perpetuation and multipotent differentiation into diverse lineages. Common mesenchymal stem cells include gingival mesenchymal stem cells (GMSCs), bone marrow mesenchymal stem cells (BMSCs), and periodontal ligament stem cells (PDLSCs), which can differentiate into osteoblasts, fibroblasts, and other cells to promote periodontal tissue repair (Gao et al., 2024; Iwasaki et al., 2022). In stem cell transplantation, hydrogels primarily serve as cell delivery platforms, with structural support as their core function; their three-dimensional porous microarchitecture provides the necessary physical anchoring sites and spatial topographical cues for cell adhesion, spreading, and proliferation (Yang et al., 2026; Zhang et al., 2025). Conversely, in growth factor applications, hydrogels function as growth factor delivery systems, aiming at spatiotemporal controlled release of bioactive molecules (Peng et al., 2024; Shan and Wu, 2024). Periodontal tissue itself constitutes a spatially graded structure extending from cementum through the periodontal ligament to alveolar bone. He et al. designed a hierarchically stratified nanofibrous scaffold capable of spatiotemporally modulating the bone-immune microenvironment, thereby promoting periodontal bone regeneration (He et al., 2024). Di Luca et al. systematically controlled three pore size ranges (<500 μm, 500–1000 μm, and 1000–1500 μm) within polycaprolactone (PCL) scaffolds, demonstrating that highly interconnected porous architectures and roughened surfaces facilitate cellular attachment and differentiation (Huri et al., 2014). Balaban et al. used rat autologous gingival mesenchymal stem cells (GMSCs) as seed cells, labeled them with green fluorescent protein (GFP), and encapsulated them in an injectable silk fibroin-chitooligosaccharide lactate (F/COS) hydrogel. They established a periodontitis animal model using the silk ligature method and systematically evaluated the therapeutic effects of this composite system in periodontal tissue regeneration. *In vitro* results showed that F/COS hydrogels effectively maintained high survival rates of GMSCs and promoted their osteogenic differentiation. *In vivo* experiments further showed that within 8 weeks of treatment, this strategy significantly reduced alveolar bone resorption, inhibited apical migration of the long junctional epithelium, and promoted regeneration of periodontal ligament structure and functional collagen fibers. In this study, the F/COS hydrogel assumes a dual role as both “scaffold and carrier”: its chemical constituents confer biocompatibility and biodegradability, while its three-dimensional porous microarchitecture provides physical anchoring sites and spatial topographical cues for cell adhesion, spreading, and intercellular communication, thereby providing a rationale for minimally invasive, autologous periodontal regeneration strategies (Balaban et al., 2024).

Furthermore, hydrogels can effectively mitigate the proteolytic degradation, burst release, and non-targeted diffusion of growth factors by confining them within the matrix, thereby extending their half-life and reducing adverse effects. Growth factor delivery via hydrogels encompasses five principal strategies-affinity-based delivery, carrier-assisted delivery, stimulus-responsive delivery, spatially structured delivery, and cell-integrated delivery-spanning applications in wound healing, tissue repair, cartilage and bone regeneration, and spinal cord injury repair (Xin et al., 2020). Wang et al. developed a blue-light-curable gelatin hydrogel for the co-delivery of a VEGF-mimetic peptide (KLT) and parathyroid hormone, which synergistically activated pro-angiogenic and pro-osteogenic pathways to accelerate bone regeneration (Wang et al., 2022). Qiu et al. designed an injectable multifunctional hydrogel system featuring a dynamic boronate crosslinked network, enabling sustained release of concentrated growth factors (CGF) and low-dose bone morphogenetic protein-2 (BMP-2) through its porous architecture while concurrently exhibiting inflammation-microenvironment-responsive release; this system effectively promoted bone repair in a periodontitis-associated bone defect model (Qiu et al., 2026).

### 3.3 Scaffolds and membrane materials

In periodontal tissue engineering, scaffold materials are required to mimic the extracellular matrix (ECM) to provide a three-dimensional (3D) microenvironment that supports cell colonization and guides organized tissue regeneration (Yamada et al., 2022). Due to their excellent biocompatibility, tunable physicochemical properties, and biomimetic capacity, hydrogels are widely regarded as ideal candidate materials for barrier membranes in guided tissue regeneration (GTR) and guided bone regeneration (GBR) (Alauddin et al., 2022). Pan et al. developed an injectable polysaccharide hydrogel scaffold incorporating nano-hydroxyapatite (nHAP); in a rat alveolar bone defect model, this scaffold completely degraded within 4 weeks, significantly promoted new bone formation without notable inflammatory response, and demonstrated excellent osteogenic activity and biosafety (Pan Y. et al., 2020). Building upon this, Krishen et al. further combined hydroxyapatite with sodium alginate to construct mechanically reinforced hydrogel membranes. These membranes integrated the biodegradability and *in situ* gelation characteristics of sodium alginate with the osteoinductive capacity of hydroxyapatite, enhancing mechanical properties while effectively promoting periodontal tissue repair, and demonstrating translational potential as functional GTR membranes (Kishen et al., 2023).

## 4 Smart hydrogels in periodontal tissue regeneration

Smart hydrogel delivery systems have garnered considerable utility as robust modalities for skeletal tissue reconstruction (Wang Xiao et al., 2025). In recent years, the development of stimuli-responsive hydrogels has attracted considerable attention (Huang et al., 2020). These hydrogels can sense changes in the local microenvironment and specifically respond to exogenous stimuli (e.g., temperature, light, mechanical forces) or endogenous signals (e.g., reactive oxygen species [ROS], specific enzyme activity), thus dynamically modulating drug release, mechanical properties, or bioactivity to optimize their therapeutic efficacy in tissue regeneration (Xu et al., 2024).

### 4.1 Exogenous stimuli

#### 4.1.1 Thermosensitive hydrogels

Thermosensitive hydrogels formulations maintain fluidic behavior under ambient conditions, enabling precise delivery to periodontal pockets via minimally invasive injection; when exposed to the oral cavity (∼37 °C), they rapidly undergo sol-gel phase transition to achieve *in situ* gelation (Vázquez-González and Willner, 2020). Below the lower critical solution temperature (LCST), these hydrogels remain in solution due to hydrogen bonding between the temperature-sensitive polymers and water, which prevents polymer chain aggregation; however, when the temperature exceeds the LCST, the polymer chains exhibit increased hydrophobicity, leading to gel formation (Bonetti et al., 2021). Based on this mechanism, Wang et al. developed a berberine-loaded thermosensitive hydrogel that undergoes gelation within approximately 3 min at body temperature. By modulating the PI3K/AKT signaling pathway, this system shows significant anti-inflammatory and pro-osteogenic effects, demonstrating promising therapeutic potential in animal models of periodontitis-induced bone destruction (Wang C. et al., 2023).

Thermosensitive hydrogels offer multiple advantages: before gelation, drugs or cells can be uniformly dispersed in the liquid precursor, which then undergoes *in situ* gelation when exposed to body temperature to form a stable polymer network, enabling efficient encapsulation; the precursor solution allows for minimally invasive delivery via conventional syringes, avoiding surgical trauma; and after gel formation, the hydrogel can adapt to conform to irregular defect sites such as periodontal pockets or bone defects, ensuring intimate contact with local tissues (Sponchioni et al., 2019). Among these, Poloxamer is currently one of the most widely used thermosensitive hydrogel matrices. Wang et al. used Poloxamer as a carrier to develop a thermosensitive hydrogel system co-loaded with egg yolk immunoglobulin (IgY) and LL-37-modified solid lipid nanoparticles (LL-37-SLNs), which was directly injected into periodontal lesion sites in its liquid state. The results demonstrated that this composite hydrogel effectively suppressed local inflammation and promoted periodontal tissue regeneration, showing significant advantages for periodontitis treatment (Wang F. et al., 2023).

#### 4.1.2 pH-sensitive hydrogels

The periodontitis-affected area exhibits a stable mildly acidic microenvironment (pH 5.5–6.5) due to inflammation, hypoxia, and bacterial metabolism, whereas healthy oral tissues maintain a neutral pH (approximately 7.2). pH-responsive hydrogels leverage the protonation or structural dissociation of functional groups under acidic conditions, triggering gelation or drug release when the pH falls below a threshold (e.g., <6.8). This enables precise identification of the lesion site and prevents drug loss from healthy sites (Xia et al., 2025). In one study, dexamethasone was encapsulated in pH-sensitive host-guest supramolecular nanoparticles, which were self-assembled from cyclodextrins (as hosts) and multivalent hydrophilic macromolecules (as guests) via non-covalent interactions; under the mildly acidic conditions of periodontitis, these nanoparticles underwent a sol-gel transition to form hydrogels *in situ*, effectively promoting drug retention and controlled release at the lesion sites, leading to enhanced local therapeutic efficacy (Li Z. et al., 2022).

**FIGURE 2:**
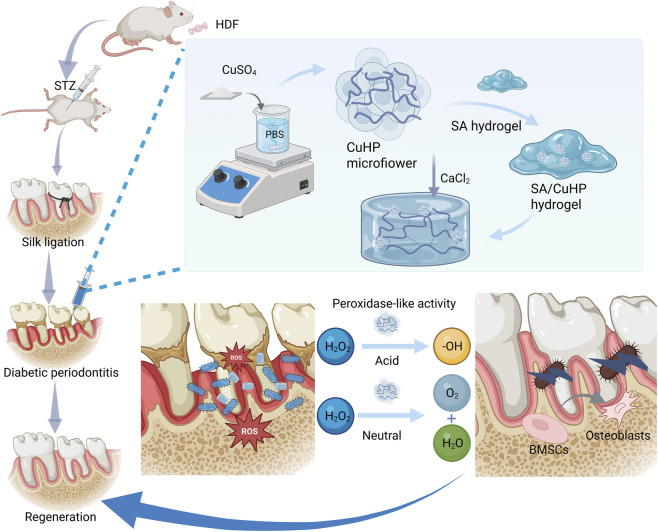
The copper hydrogen phosphate/sodium alginate (SA/CuHP) composite hydrogel possesses triple functionalities of antibacterial activity, antioxidant capacity, and osteogenic potential, capable of responding to acid-base fluctuations in the diabetic periodontitis microenvironment, thereby achieving integrated therapy of ROS regulation and bone regeneration. Reproduced with permission (Lu et al., 2025). Copyright 2025, Oxford University Press.

Recently, Lu et al. developed a pH-responsive copper hydrogen phosphate/sodium alginate (SA/CuHP) composite hydrogel (Figure 2), exhibiting triple functions of antibacterial, antioxidant, and pro-osteogenic activities. In acidic microenvironments, this material activates peroxidase-like activity to effectively eliminate pathogenic bacteria; whereas under neutral conditions, it demonstrates an intrinsic capacity to mimic catalytic decomposition kinetics, enabling the efficient quenching of superfluous reactive oxygen intermediates, mitigating the inhibition of oxidative stress on osteogenesis. Additionally, the hydrogel ensures sustained release of bioactive copper ions, synergistically promoting bone tissue regeneration. In a periodontitis animal model, this composite hydrogel significantly reduced bacterial viability, effectively suppressed alveolar bone resorption, and demonstrated excellent therapeutic efficacy along with favorable biosafety, showing potential for clinical translation (Lu et al., 2025).

#### 4.1.3 Photoresponsive hydrogels

Photoresponsive hydrogels undergo reversible changes in molecular conformation or network architecture upon exposure to specific wavelengths of light, thereby triggering drug release. By precisely modulating the duration and intensity of illumination, the drug release kinetics and dosage can be effectively controlled, achieving precise spatiotemporal therapeutic regulation (Wang Y. et al., 2023). Furthermore, owing to their excellent injectability and light-induced rapid *in situ* gelation capability, these hydrogels enable the precursor solution to penetrate deeply into periodontal pockets and irregular defect areas. Upon light irradiation, the solution rapidly gels and closely conforms to the defect morphology, overcoming the limitation of traditional preformed scaffolds, which are often incapable of adapting to complex anatomical configurations. Hu et al. developed a photocrosslinkable carboxymethyl chitosan-acrylate/antimicrobial peptide B (CMSA/Pep-B) composite hydrogel; this system undergoes sol-gel phase transition within 15 s under UV irradiation; its precursor solution shows excellent flowability, facilitating deep penetration into periodontal pockets and enabling sustained release of Pep-B to effectively suppress local inflammatory responses and reduce alveolar bone resorption (Hu Z. et al., 2023).

Gelatin methacryloyl (GelMA) is a photocurable hydrogel biomaterial that has attracted considerable attention in recent years due to its ability to effectively mimic the three-dimensional (3D) microenvironment of cells *in vivo*, and has found extensive applications across regenerative medicine and tissue reconstruction domains. Pan and colleagues demonstrated that GelMA-based hydrogel matrices could effectively entrap periodontal ligament-derived stem cells (PDLSCs), thereby facilitating the regeneration of osseous tissues. This hydrogel shows a highly porous and interconnected microstructure, providing a favorable microenvironment for stem cell adhesion, proliferation, migration, and osteogenic differentiation. Moreover, *in vivo* experiments in rats demonstrated that this scaffold significantly promoted periodontal bone repair and regeneration, showing excellent osteogenic potential and biocompatibility (Pan J. et al., 2020).

However, in infected or chronically inflamed periodontal defects, the mere provision of structural support and an osteogenic microenvironment is insufficient to achieve functional tissue regeneration; rather, materials must possess the ability to dynamically respond to pathological microenvironments. To address this, researchers developed a photoresponsive composite hydrogel using dopamine-modified sodium alginate (SA-DA) as the backbone, loaded with methylene blue (MB) and PLGA nanospheres encapsulating Semaphorin-3A (Sema3A). Upon excitation with 660 nm visible light, MB rapidly generates reactive oxygen species (ROS), achieving potent antibacterial efficacy within 14 h to create a sterile microenvironment favorable for tissue repair; subsequently, the PLGA microspheres provide sustained release of Sema3A over 28 days, inducing macrophage polarization toward the anti-inflammatory M2 phenotype, suppressing chronic inflammation and promoting osteogenic differentiation, thereby achieving sequential spatiotemporal regulation of “antibacterial-first, anti-inflammatory/osteogenesis-later” to ultimately achieve synergistic regeneration of periodontal hard and soft tissues (Xu F. et al., 2023).

#### 4.1.4 Piezoelectric hydrogels

Piezoelectric hydrogels are a class of mechanoresponsive smart materials capable of generating localized electrical charges under biomechanical stimulation derived from oral physiological activities such as mastication. The oral environment possesses unique mechanical characteristics, including periodic low-frequency mechanical loads (approximately 1–2 Hz, 0.9 MPa) and weak mechanical fluctuations. In response to this microenvironment, piezoelectric hydrogels can autonomously respond to inherent biomechanical signals, converting mechanical deformation into electrochemical signals to achieve *in situ*, on-demand electrical stimulation. Studies have demonstrated that such electrical stimulation improves mitochondrial function in damaged stem cells and enhances their osteogenic differentiation and angiogenic potential (Xu et al., 2024).

Barium titanate (BaTiO_3_, BTO) is widely used to construct such hydrogels due to its excellent piezoelectric properties. Roldán and colleagues engineered an injectable mechanoelectric hydrogel system, designated PiezoGEL, through the photocrosslinking of gelatin methacryloyl matrices homogeneously impregnated with barium titanate nanocrystals exhibiting piezoelectric functionality. Under physiological masticatory loading of the periodontium (0.9 MPa, 2 Hz), the BTO exhibits mechano-electric coupling, generating pulsed electrical signals of approximately 10 mV/mm^2^. These signals produce localized reactive oxygen species (ROS) and upregulate the bacterial oxyR oxidative stress pathway while downregulating adhesion-related genes porP and fimA, resulting in 2–3 log-reduction in bacterial viability and 41% biofilm reduction. Concurrently, these electrical signals activate ion channels in bone marrow mesenchymal stem cell (BMSC) membranes, promoting expression of early osteogenic genes including RUNX2, COL1A1, and ALP, and accelerating extracellular matrix (ECM) mineralization. *In vivo* experiments further confirmed significant reductions in periodontal pocket depth and restoration of alveolar bone height and bone volume, achieving dual antibacterial-osteogenic functions through a mechano-electro-biochemical cascade. This offers a drug-free, surgery-free intelligent biomaterial strategy for non-surgical periodontal treatment (Roldan et al., 2023).

**FIGURE 3:**
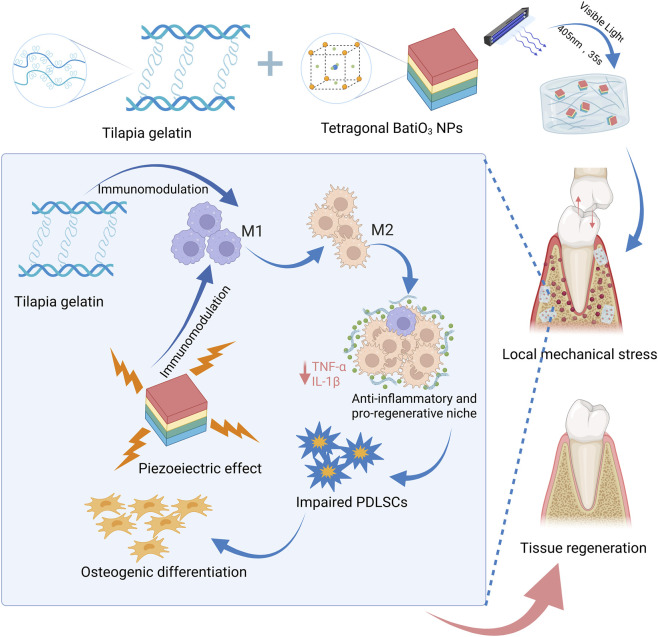
Schematic of the tilapia gelatin/tetragonal barium titanate nanoparticle (TG/BTO) piezoelectric hydrogel system. The hydrogel precursor undergoes photocrosslinking under visible light irradiation (405 nm, 35 s) to form an injectable piezoelectric scaffold. Upon placement within periodontal defects, local mechanical stress (e.g., physiological masticatory loading) activates the piezoelectric effect of BTO nanocrystals, generating mechanoelectric signals that modulate immunophenotype (suppressing M1 macrophage inflammatory cytokines and promoting M2 conversion) and enhance energy metabolism in impaired periodontal ligament stem cells (PDLSCs), thereby rescuing osteogenic differentiation and driving tissue regeneration in inflammatory periodontal bone defects. Reproduced with permission (Liu X. et al., 2024). Copyright 2024, Elsevier.

Furthermore, Liu et al. developed an injectable piezoelectric hydrogel based on tilapia gelatin and incorporating tetragonal barium titanate nanoparticles (BTO NPs). The hydrogel precursor is photocrosslinked under visible light (405 nm, 35 s) to form a stable scaffold prior to implantation. Following placement in periodontal defects, physiological mechanical stimulation (e.g., simulated mastication at 0.9 MPa, 2 Hz) activates the piezoelectric effect of BTO NPs, generating mechanoelectric signals that significantly increase intracellular ATP levels in impaired periodontal ligament stem cells (PDLSCs) under inflammatory conditions, thereby restoring their osteogenic differentiation potential (Figure 3). Simultaneously, mechanoelectric signaling originating from piezoelectric elements operated in concert with the endogenous immunomodulatory attributes of tilapia-derived gelatin. This cooperative interaction drove the phenotypic transition of macrophages from the classically activated M1 state to the alternatively activated M2 state, consequently establishing a regenerative immune niche conducive to tissue restoration. *In vivo* investigations revealed that this mechanoelectric hydrogel construct substantially enhanced *de novo* osseous formation within a rodent model of periodontitis-induced skeletal defects. The therapeutic efficacy was attributed to the synergistic orchestration of mechanotransductive, electroactive, and immunomodulatory signaling pathways (Liu X. et al., 2024).

### 4.2 Endogenous stimuli

#### 4.2.1 Matrix metalloproteinase (MMP)-responsive hydrogels

**FIGURE 4:**
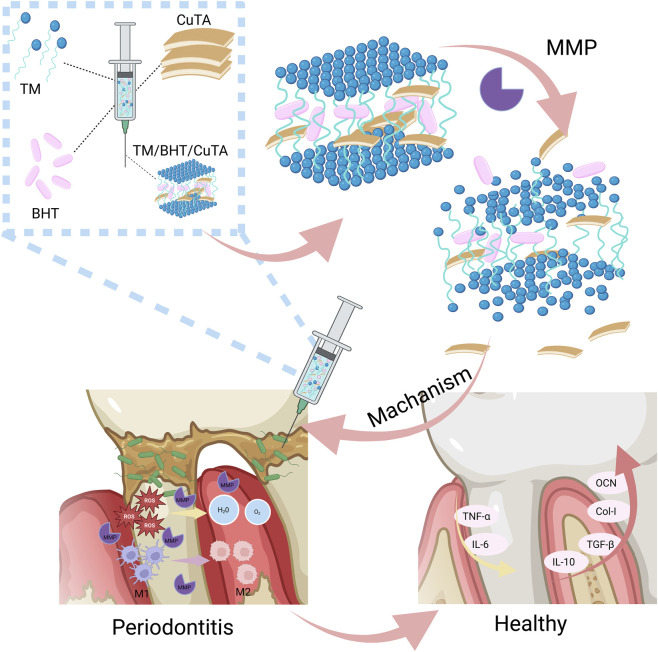
The TM/BHT/CuTA composite hydrogel system inhibits matrix metalloproteinase (MMP) activity, downregulates TNF-α/IL-6 and upregulates IL-10/TGF-β, thereby driving macrophage polarization from M1 to M2 phenotype, reducing ROS levels, restoring Col-I and OCN expression, and thereby achieving the reversal from periodontitis to healthy periodontal tissue. Reproduced with permission (Xu Y. et al., 2023). Copyright 2023, American Chemical Society.

Matrix metalloproteinase (MMP)-responsive delivery systems have been established as intelligent strategies for targeting inflammatory microenvironments. Within the pathological milieu of periodontal inflammation, matrix metalloproteinases (MMPs) exacerbate deterioration of tooth-supporting structures and mediate alveolar bone loss through proteolytic ECM breakdown, pro-inflammatory mediator activation, and osteoclastogenic signaling amplification. These interconnected processes perpetuate a self-sustaining cycle characterized by progressive matrix catabolism, escalating inflammatory responses, and irreversible skeletal damage (Lin et al., 2024). Given the critical role of MMPs in the pathological progression of periodontitis, researchers have recently focused on developing intelligent delivery systems capable of responding to microenvironments with elevated MMP expression, thereby achieving precise release of therapeutic agents and inflammatory regulation. Xu et al. constructed a composite hydrogel incorporating glyceryl monostearate/2,6-di-tert-butyl-4-methylphenol (TM/BHT) and copper-tannic acid coordination nanosheets (CuTA NSs), designed to promote bone tissue regeneration under pathological conditions of periodontitis. This hydrogel is responsive to MMPs and can be specifically degraded by highly expressed MMPs in the chronic periodontal inflammatory environment, thereby triggering on-demand release of CuTA nanozymes. The liberated bioactive moieties orchestrate a dual-functional response: driving phenotypic switching of macrophages toward alternatively activated states while concomitantly enhancing transcriptional activity of genes governing osseous differentiation. These coordinated events facilitate the restoration of functional integrity within compromised periodontal structures (Xu Y. et al., 2023) (Figure 4).

However, in bone defects associated with systemic metabolic disorders such as diabetes, the inflammatory microenvironment is characterized not only by elevated MMP expression but also by the presence of multiple pathological signals, including ROS accumulation and hyperglycemic fluctuations, rendering single-responsive mechanisms insufficient for dynamic adaptation to complex disease progression. To address this, Li et al. developed an HIB dual-network hydrogel by embedding drug-loaded gelatin nanoparticles within a ROS/glucose-responsive PVA-TSPBA network, achieving a “diagnosis-therapy” cascade mechanism: the MMP-2/9 cleavage sites within the gelatin matrix serve as critical triggering elements recognized and hydrolyzed by highly expressed MMP-9 in the diabetic microenvironment, leading to degradation and detachment of the gelatin network to release IL-10; simultaneously, the PVA network undergoes cleavage upon ROS/hyperglycemia stimulation, enabling delayed release of BMP-2, thereby creating a spatiotemporal program of “anti-inflammation first, osteogenesis second”. MMP-responsive degradation not only precisely initiates the immunomodulatory phase but also disrupts physical crosslinking between nanoparticles, synergistically reducing hydrogel integrity to accelerate subsequent osteogenic factor release, ultimately remodeling the mitochondrial antioxidant system of macrophages to achieve efficient bone regeneration in diabetic bone defects under hyperglycemic conditions (Li D. et al., 2022).

#### 4.2.2 Reactive oxygen species (ROS)-responsive hydrogels

**FIGURE 5:**
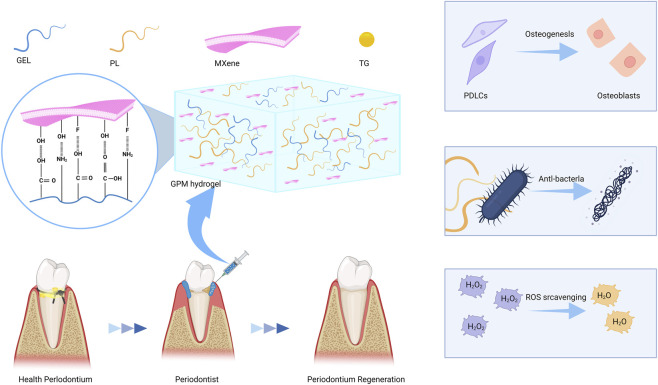
Periodontal regeneration strategy based on MXene/PL composite GPM hydrogel: Through ROS scavenging and modulation of the chemical microenvironment (OH, H/O), osteogenic differentiation and bone formation are promoted, synergistically activating PDLCs with signaling molecules such as TG, thereby achieving periodontal tissue regeneration and offering a novel approach for clinical treatment. Reproduced with permission (Yu et al., 2024). Copyright 2024, American Chemical Society.

Oxidative stress-activated hydrogel systems exhibit considerable promise for the management of periodontal disease-induced alveolar bone deficiencies. Under inflammatory conditions, excessive ROS accumulation disrupts the redox balance, induces local oxidative stress, and subsequently accelerates destruction of periodontal supporting tissues (Sczepanik et al., 2020). To address this pathological feature, Yu et al. designed a ROS-responsive hydrogel by integrating Ti_3_C_2_T_x_ MXene nanosheets and poly-L-lysine (PL) into a gelatin matrix, constructing a multifunctional injectable system (GPM) with antibacterial, antioxidant, and pro-osteogenic functionalities. Abundant -OH/-O groups on MXene surfaces directly undergo redox reactions with ROS (e.g., H_2_O_2_, ·O_2_^−^) and are depleted during this process, thereby rapidly scavenging excessive ROS in the inflammatory microenvironment, significantly mitigating oxidative stress-induced damage to human periodontal ligament cells (hPDLCs), inhibiting overactivation of inflammatory signaling pathways such as NF-κB, and consequently reducing expression of pro-inflammatory factors such as IL-1β. Concurrently, downregulation of ROS levels acts synergistically with the potent antibacterial effects mediated by PL to effectively control local infection and inflammatory infiltration; based on this anti-inflammatory and antioxidant microenvironment, MXene further promotes osteogenic differentiation of hPDLCs and upregulates expression of osteogenesis-related genes including RUNX2, ALP, and OCN. *In vivo* experiments confirmed that the GPM hydrogel significantly reduces ROS accumulation in periodontal tissues, suppresses osteoclastic activity, restores alveolar bone height and volume, and promotes organized regeneration of junctional epithelium, ultimately achieving functional repair of periodontal hard and soft tissues (Yu et al., 2024) (Figure 5).

Periodontal pathologies concurrent with systemic metabolic derangements, exemplified by diabetes mellitus, reactive oxygen species (ROS) act not only as inflammatory mediators but also synergize with hyperglycemia to disrupt the homeostasis of redox status, inflammatory responses, and bone metabolism, thus requiring smart materials with enhanced antioxidant and immunomodulatory capabilities. Recently, Ge and colleagues engineered an oxidative stress-activated, syringe-deliverable hydrogel matrix (GOE1) exhibiting autonomous structural restoration capabilities. This construct was functionalized with nanoparticles encapsulating epigallocatechin-3-gallate complexed with chlorhexidine acetate (EGCG/CHX), enabling precision intervention for periodontal pathologies concurrent with metabolic dysregulation. Within hyperglycemic microenvironments, excessive ROS induce oxidative damage and activate pro-inflammatory pathways, including nuclear factor-kappa B (NF-κB) and interleukin-17 (IL-17), thereby driving a pathological cycle of “oxidative stress-chronic inflammation-alveolar bone resorption”. Leveraging the polyphenolic architecture of epigallocatechin gallate, GOE1 exhibits multifaceted immunomodulatory capabilities: efficient neutralization of reactive oxygen intermediates, mitigation of oxidative insults, and fine-tuning of inflammatory cascades. This green tea-derived construct promotes phenotypic switching of macrophages toward alternatively activated phenotypes, concomitantly dampening the secretion of TNF-α and IL-6 while upregulating the biosynthesis of resolution-promoting mediators such as IL-10 and Arg1. Animal experiments demonstrated that this hydrogel significantly reduced ROS levels in periodontal tissues, reduced inflammatory cell infiltration and collagen destruction, effectively inhibited alveolar bone resorption, and promoted tissue regeneration, offering a promising ROS-responsive targeted antioxidant therapeutic approach for diabetic periodontitis (Ge et al., 2025).

## 5 Other hydrogel systems

### 5.1 Probiotic hydrogels

The utilization of probiotic formulations for modulating oral microbial communities has garnered substantial interest within the scientific community over recent years. Their underlying mechanisms primarily include enhancing colonization of commensal microbiota, suppressing the growth of pathogenic microorganisms, and disrupting the formation of oral pathogenic biofilms and virulence factor expression through the secretion of antimicrobial compounds (e.g., bacteriocins). An interventional trial performed in individuals diagnosed with type 1 diabetes mellitus assessed the impact of toothpaste incorporating phytochemical constituents alongside probiotic formulations on both periodontal status and metabolic biomarkers. The findings revealed that following probiotic intervention, subjects exhibited marked amelioration across diverse periodontal clinical indices, encompassing diminished sulcular penetration depths, decreased dental plaque scores, improved periodontal attachment heights, and reduced periodontal bleeding tendency, along with significant improvements in glycated hemoglobin (HbA1c) levels, suggesting that probiotics may contribute to glycemic control through the regulation of oral microbial homeostasis (Butera et al., 2022; Cannizzaro et al., 2023).

Previous studies have highlighted the potential of oral microbiota modulation in the periodontal-systemic health link; however, conventional probiotics exhibit poor survival in inflammatory conditions and limited functionality, making them inadequate to address the complex pathological features of diseases such as diabetic periodontitis. To address this, researchers have further developed synthetic nanosystems with probiotic-mimetic functions to achieve more stable and efficient multitargeted therapy. Recently, researchers developed an ultrasound-responsive polyphenol-based nanoprobiotic hydrogel (Fe-TCPP-DA-Zn/ODG) that significantly reduced periodontal tissue damage associated with diabetic periodontitis through multiple mechanisms including antimicrobial activity, antioxidant effects, immunomodulation, and microbial homeostasis. Furthermore, by modulating the oral-brain axis, this system effectively improved associated cognitive deficits and emotional dysregulation, thereby offering a novel strategy for dual-targeted therapy of periodontal and neurological disorders (Yang et al., 2025).

### 5.2 Metal-organic framework (MOF)-based hydrogels

Porous coordination polymers, commonly designated as metal-organic frameworks (MOFs), have garnered substantial interest across biomedical applications in recent years. This attention stems from their architecturally adjustable frameworks, exceptional resistance to chemical degradation, and sustained pathogen-inhibitory efficacy (Yu et al., 2025). Compared with conventional antimicrobial agents, MOF-based materials exhibit distinct advantages: they not only facilitate controlled release and extended activity of antimicrobial components but also enable precise design at the molecular level to enhance their performance as antimicrobial carriers, and exhibit potential for synergistic effects with other antimicrobial agents (Pettinari et al., 2021).

**FIGURE 6:**
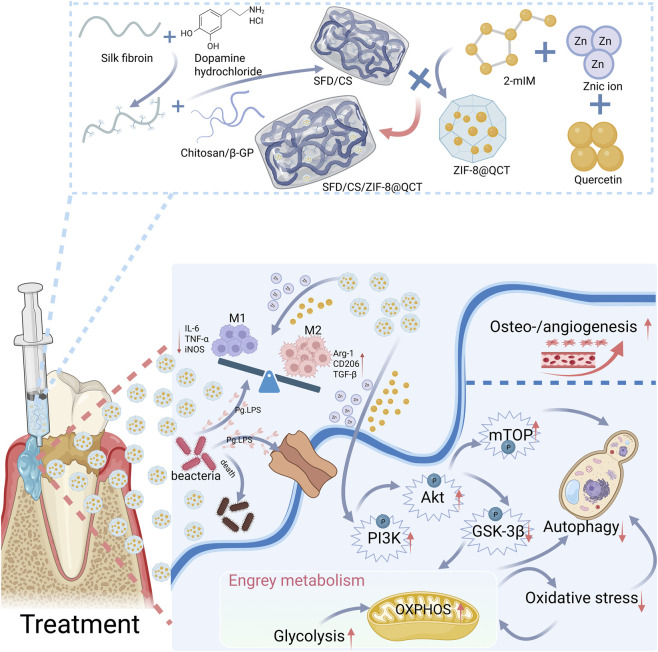
Graphical representation of the SFD/CS/ZIF-8@QCT hybrid hydrogel system: This multifunctional construct facilitates alveolar bone restoration in periodontal pathologies via antimicrobial efficacy, hemorrhage control, immune microenvironment modulation, osteogenic and angiogenic stimulation, and enhanced progenitor cell mobilization. Reproduced with permission (Yang et al., 2024). Copyright 2024, Elsevier.

Building on these findings, researchers have incorporated MOFs into hydrogel systems for periodontitis treatment, with the resultant composite materials demonstrating both excellent antibacterial efficacy and good biocompatibility. For instance, Yang et al. recently developed a novel injectable MOF-functionalized hydrogel (SFD/CS/ZIF-8@QCT); this strategy utilizes metal–organic frameworks (MOFs) to construct a multifunctional, injectable thermosensitive hydrogel for periodontal tissue regeneration within the pathological microenvironment of periodontitis. In this system, ZIF-8, acting as a pH-responsive MOF, not only enables stimuli-responsive release of zinc ions with antibacterial and pro-regenerative properties but also efficiently encapsulates the natural flavonoid compound quercetin (QCT), forming ZIF-8@QCT hybrid nanoparticles (Figure 6). This MOF-based carrier markedly enhanced the multiple biological functions of the hydrogel: providing effective antibacterial activity via disruption of bacterial cell membranes and inhibition of virulence factor expression; mediating immunomodulation by regulating macrophage polarization; while reducing oxidative stress, inhibiting abnormal autophagy, and synergistically promoting osteogenic differentiation and angiogenesis (Yang et al., 2024).

These studies have shown that MOFs, as functional building blocks, can impart stimuli-responsive behavior and multifunctional synergistic properties to hydrogels; furthermore, by modulating the metal nodes and organic linkers, offering potential to further optimize their antioxidant, anti-inflammatory, and osteogenic properties. Recently, a study developed an injectable self-healing hydrogel (CSBDX@MOF) incorporating magnesium-gallate MOFs (Mg-GA MOFs), and validated its therapeutic potential in periodontal bone regeneration through *in vitro* and *in vivo* experiments: *In vitro* experiments demonstrated that the MOF significantly eliminated ROS, inhibited the growth of periodontal pathogens, induced macrophage polarization toward the M2 phenotype, and promoted osteoblast differentiation and mineralization; in addition, rat periodontitis models confirmed that CSBDX@MOF effectively reduced inflammatory cell infiltration, decreased bone loss biomarker expression, significantly increased bone volume fraction (BV/TV), and reconstructed alveolar bone height, suggesting that MOFs, as the key active components of this system, play a key role in antibacterial activity, anti-inflammatory effects, immune regulation, and bone regeneration (Luo et al., 2024).

## 6 Conclusion and perspectives

The design and application of hydrogels for periodontal tissue regeneration must be grounded in a meticulous understanding of biocompatibility. Biocompatibility is not an inherent property of a material but rather a highly context-dependent, multifaceted concept that depends on a delicate balance of chemical composition, crosslinking chemistry, degradation byproducts, surface bioactivity, substrate stiffness, and other physicochemical characteristics, ultimately determining whether an appropriate host response and functional integration can be achieved within the specific periodontal microenvironment. Based on this design philosophy, coupled with their tunable three-dimensional microarchitecture and synergistic co-delivery capacity with antimicrobial agents, hydrogels exhibit significant promise for periodontal tissue regeneration therapy, positioning them as a promising research frontier in this field. Their primary applications are as drug delivery systems for localized sustained delivery of antibacterial and anti-inflammatory agents, delivery vehicles for cells and growth factors to promote tissue repair, and scaffolds or engineered membranes to provide mechanical support for periodontal tissue regeneration. Recently, stimuli-responsive hydrogels responsive to stimuli in the periodontal microenvironment have facilitated on-demand, controlled drug delivery at the infection site. Investigators have successfully incorporated antimicrobial compounds, immunomodulatory agents, and tissue-inductive biomolecules within three-dimensional hydrogel networks. This strategic integration yields multifunctional therapeutic constructs that concurrently combat pathogenic invasion, resolve inflammatory cascades, and promote functional tissue neogenesis.

However, despite their promise, the clinical translation of hydrogels for periodontal tissue regeneration still faces significant hurdles: First, the periodontal tissue structure is highly complex, consisting of distinct tissues including cementum, periodontal ligament (PDL), and alveolar bone; however, existing hydrogels primarily enable single-tissue regeneration, thereby hindering simultaneous repair of multiple components; Second, although hydrogels possess stimuli-responsive properties, their sensing of and response to the dynamic oral microenvironment lack precision, compromising drug delivery accuracy; Third, most hydrogels possess insufficient mechanical strength, making them prone to deformation or detachment in the complex mechanical environment of the oral cavity, and failing to resist physiological forces such as masticatory forces; Fourth, terminal sterilization and long-term storage stability remain critical barriers to clinical translation. Hydrogels are inherently sensitive to conventional sterilization methods-autoclaving, gamma irradiation, and ethylene oxide treatment can each induce undesirable alterations in chemical structure, mechanical integrity, swelling behavior, and biocompatibility (Galante et al., 2018), and the outcome is highly dependent on the specific hydrogel composition and sterilization parameters, making it virtually impossible to establish universal sterilization guidelines (Bento et al., 2023). Furthermore, maintaining the physicochemical and functional stability of hydrogels during extended storage is essential for regulatory approval and practical clinical deployment (Wang Xin et al., 2025), yet this aspect remains understudied for most smart hydrogel systems designed for periodontal applications.

In summary, hydrogel matrices function as versatile therapeutic carriers for the management of periodontal pathologies, offering a viable translational strategy for clinical intervention. However, hydrogels remain in the preclinical stage in periodontal regeneration without widespread clinical adoption; future research should optimize *in vivo* study designs, rigorously assess their long-term safety and therapeutic efficacy, and prioritize improving the mechanical properties, precision of environmental responsiveness, and capacity for coordinated multi-tissue regeneration of these materials, to accelerate their clinical translation and provide more efficient and precise regenerative strategies for this disease.
